# Clinical assessment of Shenfu injection loading in the treatment of patients with exacerbation of chronic heart failure due to coronary heart disease: study protocol for a randomized controlled trial

**DOI:** 10.1186/s13063-015-0729-7

**Published:** 2015-05-21

**Authors:** Chunxiang Liu, Yazhu Hou, Xianliang Wang, Zhiqiang Zhao, Zhi Liu, Jingbo Zhai, Jingyuan Mao, Hongcai Shang

**Affiliations:** Evidence-based Medicine Center, Tianjin University of Traditional Chinese Medicine, 312 Anshan Western Road, Nankai District, Tianjin, 300193 China; Cardiovascular Department of The First Teaching Hospital of Tianjin University of Traditional Chinese Medicine, 314 Anshan Western Road, Tianjin, Nankai District, Tianjin, 300193 China; Tianjin University of Traditional Chinese Medicine, 312 Anshan Western Road, Nankai District, Tianjin, 300193 China; Key Laboratory of Chinese Internal Medicine of Ministry of Education and Beijing, Dongzhimen Hospital, Beijing University of Chinese Medicine, Beijing, 100700 China

**Keywords:** Traditional Chinese medicine, Acute heart failure, Shenfu injection

## Abstract

**Background:**

Acute exacerbation is a common cause of hospitalization in patients with chronic heart failure, and coronary heart disease is the most common cause. Shenfu injection, a Traditional Chinese Medicine injection, widely used in the adjuvant treatment of patients with acute exacerbation of chronic heart failure, shows some treatment effect in improving the symptoms and the quality of life, but it lacks the rigorous clinical evaluation of research reports. This paper describes the protocol for the clinical assessment of Shenfu injection loading in the treatment of patients with acute exacerbation of chronic heart failure.

**Methods:**

This protocol adopts the design of a prospective, randomized, multicenter, blind imitation, placebo-controlled trial to assess the efficacy and safety of Shenfu injection loading in the treatment of patients with acute exacerbation of chronic heart failure due to coronary heart disease. The research will be carried out in 12 hospitals in China and is expected to enroll 160 inpatients with acute exacerbation of chronic heart failure due to coronary heart disease (*yang* and *qi* deficiency syndrome). On the basis of the conventional therapy of western medicine, patients will be randomized to either the treatment group (100 ml 5% glucose injection + 50 ml Shenfu injection) or the control group (150 ml 5% glucose injection) for 7 ± 1 days and follow-up for 28 ± 3 days. The primary outcomes are New York Heart Association cardiac function classification and Traditional Chinese Medicine syndromes. The secondary outcomes are left ventricular ejection fraction, brain natriuretic peptide level, Lee’s heart failure score, 6-minute walking distance, and the incidence and readmission rate of cardiovascular events (including the emergency rate due to acute exacerbation of chronic heart failure).

**Discussion:**

This trial will assess the effect of loading Shenfu injection in the treatment of patients with acute exacerbation of chronic heart failure caused by coronary heart disease (*yang-qi* deficiency syndrome) on the symptoms and signs of heart failure, exercise tolerance, and other aspects, and observe its influence on the short-term prognosis with follow-up. The results of the study will provide clinical research evidence for application of Shenfu injection in the treatment.

**Trial registration:**

This trial was registered on 26 December 2012 at the Chinese Clinical Trials Register (Identifier: ChiCTR-TRC-12002857).

**Electronic supplementary material:**

The online version of this article (doi:10.1186/s13063-015-0729-7) contains supplementary material, which is available to authorized users.

## Background

Chronic Heart failure (CHF) is a chronic progressive disease, the terminal stage of various cardiovascular diseases and a cardiac dysfunction syndrome. The condition of CHF patients will remain stable after treatment, but will also dramatically worsen under the influence of various factors (such as infection, rapid ventricular rate arrhythmia, and so forth) and appears as acute decompensated CHF (that is, acute exacerbation of CHF (AECHF)) [1].

The first guideline for diagnosis and treatment of acute heart failure (AHF) in China was promulgated in March 2010 [2]. “AHF” referred to sudden onset or acute exacerbation of the original CHF, expanding the scope of AHF, and acute decompensation of CHF was included. *The diagnosis and treatment guideline for heart failure of China in 2014* [3] indicates that AHF is the main cause of hospitalization in patients whose age has exceeded 65 years, most of them with acute exacerbation based on the original CHF.

A study [4] which retrospectively analyzed 10,714 hospitalized cases retrieved from 42 hospitals in China over three different time periods (the 1980s, 1990s, and 2000s) showed that patients hospitalized for CHF accounted for about 16.3 to 17.9% of those with cardiovascular disease. The primary cause of CHF was coronary heart disease (CHD), most of the cardiac function of HF was grade III(42.5 to 43.7%),and the main cause of death in heart failure patients was left heart failure (59%).

The treatment for AECHF, according to the guideline [3], which is the same as the treatment for AHF, includes: general treatment (such as oxygen therapy, controlling the intake and output, and so forth); medical treatment (such as diuretics which can reduce cardiac load and improve symptoms); treatment with drugs or/and non-drug therapy for the main disease and complications depending on patient’s conditions. The symptoms of AECHF can be alleviated over a short period using simple western medicine treatment, shown by improvement in heart function, hemodynamic indices and biological indices. Presently, Traditional Chinese Medicine (TCM) treatment for AECHF is still secondary to western medicine, and TCM injection, such as Shenfu injection (SFI) [5] and Shenmai injection [6] ,are commonly used by TCM syndrome.

A retrospective study [7] showed that the main TCM syndrome of CHF was deficiency of *qi*, with *yang* or/and *yin*. The result of a TCM syndrome survey [8] showed that CHF was divided into two types: *qi-yang* deficiency with blood stasis and/or phlegm retention; and *qi-yin* deficiency with blood stasis and/or phlegm retention. The pattern of syndrome change [9] was *qi* deficiency → *qi* deficiency, *qi-yin* deficiency →*qi-yang* deficiency with blood stasis and/or phlegm retention.

Based on the ancient prescription of Shenfu Decoction, SFI is composed of the extracts of red ginseng and Radix Aconiti Lateralis Preparata using modern technology. The active ingredients include Ginsenoside and Aconitine [10], which have the function of supplementing *qi*, avoiding collapse and reviving *yang*. SFI was an officially approved product in 1987 (approval code: certification number Z20043117; norm: 50 ml per bottle). Many clinical reports indicated that SFI was convenient for emergency use and had the advantages of rapid effect [11], strengthening the heart, improving heart function, protecting vascular endothelial cells, regulating immune function, anti-inflammation and improving ability of hypoxia tolerance [12].

### Objective

The objective of this trial is to observe the influence of heart function and TCM syndrome and to assess the short-term effectiveness and safety in patients with AECHF due to CHD (*yang-qi* deficiency syndrome) treated with SFI loading.

## Methods

### Study design

**Figure 1:**
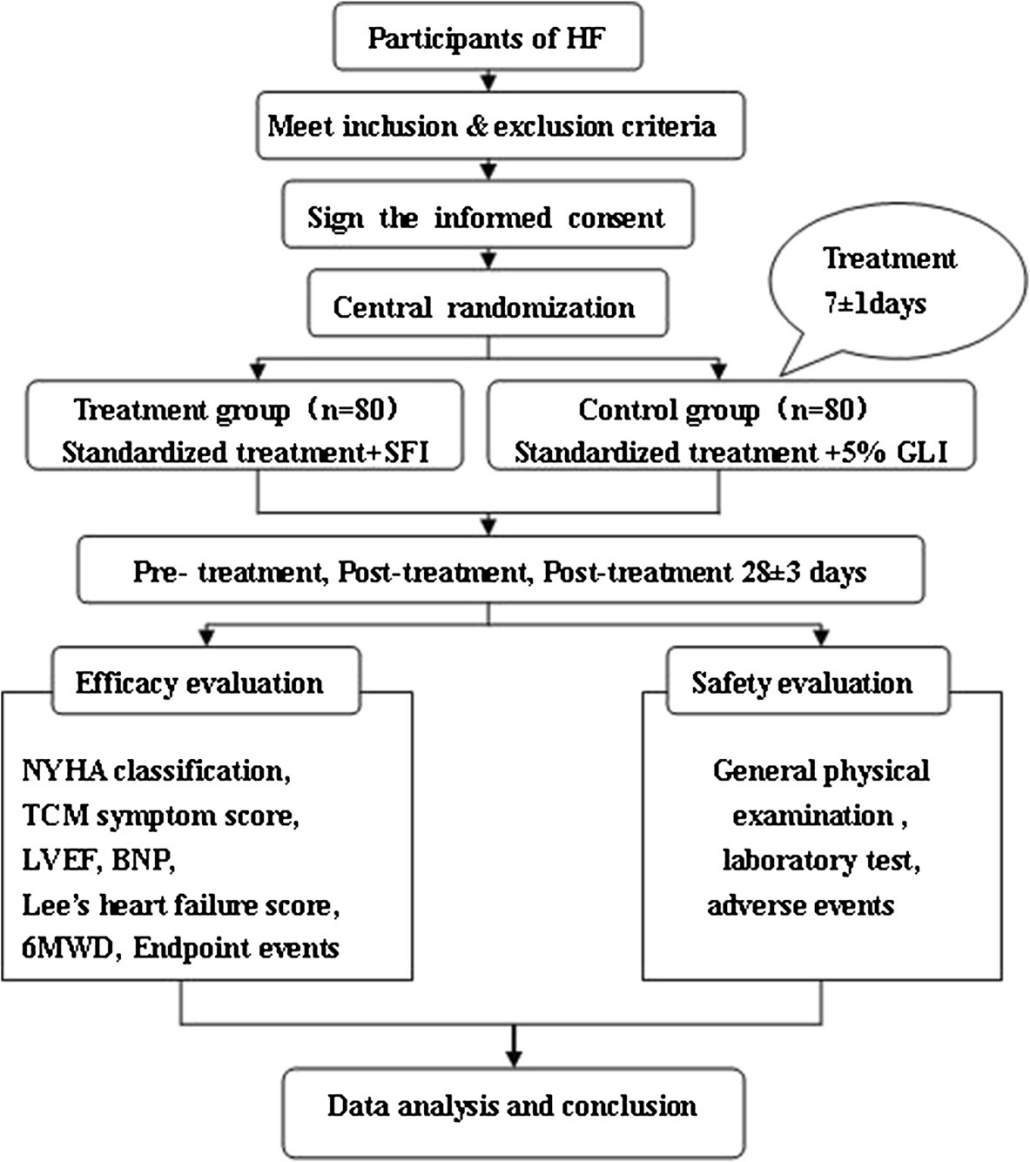
**Schedule of evaluations.** The overall plan of the trial and each step that needs to be performed is listed. CHF, chronic heart failure; SFI, Shenfu injection; GLI, glucose injection; NYHA, New York Heart Association; TCM, Traditional Chinese Medicine; LVEF, left ventricular ejection fraction; BNP, brain natriuretic peptide; 6MWD, 6-minute walking distance.

This study is a prospective, randomized, multicenter, blind imitation, placebo-controlled trial. In order to avoid regional differences, participating centers include 12 hospitals located in the north and south of China. All cases included are hospitalized patients. Totally 160 subjects are randomly divided into treatment and control groups. The specific scheme is shown in Figure 1.

### Sample size

This is a clinical pilot trial; therefore, the total sample size of this study is determined to be 160 (during the trial, the expulsion rate is controlled within 20%) on expert advice. The treatment group and the control group have the same number of cases (that is, there are 80 cases in each group).

### Diagnostic criteria

#### Diagnostic criteria for coronary heart disease

The diagnostic criteria for CHD were: 1) history of myocardial infarction, with or without revascularization (percutaneous coronary intervention or coronary artery bypass grafting) treatment; and/or 2) coronary angiography or computed tomography coronary angiography confirmation of stenosis above 50% of at least one major branch of the coronary artery luminal diameter, with or without revascularization. A patient with one of these criteria is to be diagnosed with CHD.

#### Diagnostic criteria for acute exacerbation of chronic heart failure

According to *The diagnosis and treatment guideline for CHF of China in 2007* [13], *The diagnosis and treatment guideline for AHF of China in 2010* [2], *Executive Summary: HFSA 2010 Comprehensive Heart Failure Practice Guideline* [14], the diagnostic criteria for AECHF were: 1) having a medical history, symptoms or signs of heart disease, and the main symptoms and signs of dyspnea, fatigue, fluid retention (edema), jugular venous engorgement, and so forth; and 2) left ventricular ejection fraction (LVEF) ≤40% by the Simpson method.

#### Diagnostic criteria for cardiac function classification

Referring to the New York Heart Association updated version in 2005 [15], the criteria for heart functional class III were that the patients suffer from heart disease, and physical activity is significantly restricted (fatigue, palpitation, dyspnea or angina pectoris when the activity amount is less than normal), and for heart functional class IV that the patients suffer from heart disease, and physical activity is completely lost (symptoms of heart failure or angina pectoris are present even at rest, and any slight physical activity can make symptoms worse, for example dyspnea and fatigue).

#### The standardization of Traditional Chinese Medicine differentiation

According to *The Guidelines for Clinical Research of new TCM drugs (2002)* [16,17], the primary signs and symptoms are palpitation, shortness of breath (dyspnea), and fatigue. The secondary signs and symptoms are spontaneous perspiration, disinclination to talk, wheezing, cough, chest stuffiness (pain), loss of appetite, abdominal distension, aversion to cold, cold limbs, and edema. In addition, the tongue and pulse presentation is of a pale and dark tongue, which is fat or with indentation, and the pulse is sunken, slow or quick, or knotted and intermittent.

A patient must meet all the primary symptoms and at least two of the secondary symptoms listed above, consulted with the tongue and pulse presentation, to be diagnosed with *yang-qi* deficiency syndrome.

### Inclusion criteria

1. Patients aged 40 to 79 years;
2. Diagnosis of CHD, AECHF;
3. New York Heart Association Class II to IV;
4. TCM diagnosis of *yang- qi* deficiency syndrome;
5. Provision of written informed consent by participants or surrogates.

### Exclusion criteria

1. Patients with acute coronary syndrome (cardiac troponin T/ cardiac troponinI, creatine kinase-MB isoenzyme as screening index, acute myocardial infarction within 6 months, revascularization within the past 6 months or planned for revascularization within 1 week after inclusion, cardiogenic shock, lethal arrhythmia, cardiomyopathy, rheumatic valvular heart disease, myocarditis, constrictive pericarditis, pulmonary embolism, and so forth.
2. Severe hepatic, renal insufficiency (alanine transaminase ≥3 times the normal upper limit, creatinine ≥3 mg/dL;
3. Combined with the endocrine system, hematopoietic system and other severe primary diseases;
4. Preparing for pregnancy within 3 months, pregnant, or breast-feeding women;
5. Patients with psychosis;
6. Patients with an allergic constitution, known sensitivity to the study drugs or their ingredients;
7. Participation in other clinical trials within 3 months;
8. Researchers estimate that the survival period of patient is not more than 3 months;
9. Researchers estimate that the patient cannot complete or comply with the requirements of this study.

### Randomization

This study will use the central randomization system of the Tianjin Institute of Clinical Evaluation to achieve the distribution of patients and drugs.

The central randomization system will adopt age (40 to 49, 50 to 59, 60 to 69, and 70 to 79 years) and cardiac function classification as the central random control factors to dynamically and randomly allocate the participants, keeping a balance between the two groups to avoid selection bias; it will also monitor the real-time drug distribution and inventory information of each center in order to timely supply drugs and effectively avoid drug excess, saving the resources of manpower and materials.

### Blindness

Because SFI is a colored transparent liquid, nurses use the disposable light-proof infusion cover to effectively block the test drug and 5% glucose injection (GLI) in order to reduce bias. This study will blind doctors, evaluators, patients and statistical experts, but not the nurses.

### Interventions

According to *The diagnosis and treatment guideline for AHF of China in 2010* [2] and *The diagnosis and treatment guideline for CHF of China in 2007* [13], doctors will choose medications based on conventional Western medicine treatment, and are prohibited from using other TCM in addition to the test drug. Combined with the cardiac function of patients, the basal therapy for CHF and AECHF are as follows: diuretics (furosemide injection for the first 7 ± 1 days and oral tablet during the follow-up period), angiotensin-converting enzyme inhibitors or angiotensin receptor blockers, β-blockers, aldosterone antagonists, digitalis, anti-platelet drugs, statin drugs, and so forth.

In order to reduce the bias of the test results, the control group is treated with 150 ml 5% GLI. The injection volume for the control group is therefore the same as the treatment group. All patients need to have a fasting blood glucose test and diabetic patients have to adjust the dose of insulin before participating in the trial.

The test drug is used as follows. For the treatment group: 50 ml SFI dissolved in 100 ml 5% GLI delivered via intravenous drip within 150 minutes, once a day; for the control group: 150 ml 5% GLI via intravenous drip within 150 minutes, once a day; both over 7 ± 1 days as a course.

### Outcome measurements

#### Primary outcomes

The primary outcomes of the study include the effective rate on the improvement of heart functional class and TCM syndrome. TCM syndrome is evaluated by grading the quantitative score according to the primary symptoms and secondary symptoms. Two indices, which are measured before and after treatment, are simple measurements used to judge the degree of CHF. The severity of heart failure symptoms has a poor correlation with ventricular function, but is clearly associated with survival rate [3].

#### Secondary outcomes

Combined with physical and chemical examinations pre- and post-treatment, changes in objective indices are used to evaluate the therapeutic effect.

LVEF and other echocardiographic indices reflect left ventricular function. In view of the correlation between left ventricular volume, LVEF and angiography on autopsy, the trial uses the modified Simpson method to measure echocardiographic indices, and the relevant standard operating procedure is presented in the investigator’s manual.

The plasma brain natriuretic peptide (BNP) level can be used as an auxiliary method to evaluate the treatment efficacy. Test instruments(Biosite Triage ® MeterPlus) will be unified to avoid measurement error.

The trial will combine the symptoms and signs with X-ray cardiac telephotography examination for assessment of Lee’s heart failure score.

Six-minute walking distance is measured within 24 hours after inclusion and also after treatment to evaluate the change in exercise tolerance. At the same time, the trial also observes the occurrence of end-point events, and analyzes the incidence and readmission rate for cardiovascular events.

#### Safety outcomes

Safety is assessed by vital signs, laboratory examinations and adverse events. Vital signs include blood pressure and heart rate. Laboratory examinations include routine blood and urine, routine stool and fecal occult blood, hepatorenal function, electrolytes and electrocardiogram. The purpose of examining routine stool and fecal occult blood is to prevent gastrointestinal hemorrhage because of the treatment with antiplatelet aggregation, anticoagulant drug. The purpose of examining electrolytes (K^+^, Na^+^, Cl^−^) is to prevent electrolyte disorder for heart failure treatment with diuretics.

Adverse events will be recorded during the treatment. The prognosis of adverse events will also be observed until the adverse reactions disappear or are relieved.

### Data collection

**Figure 2:**
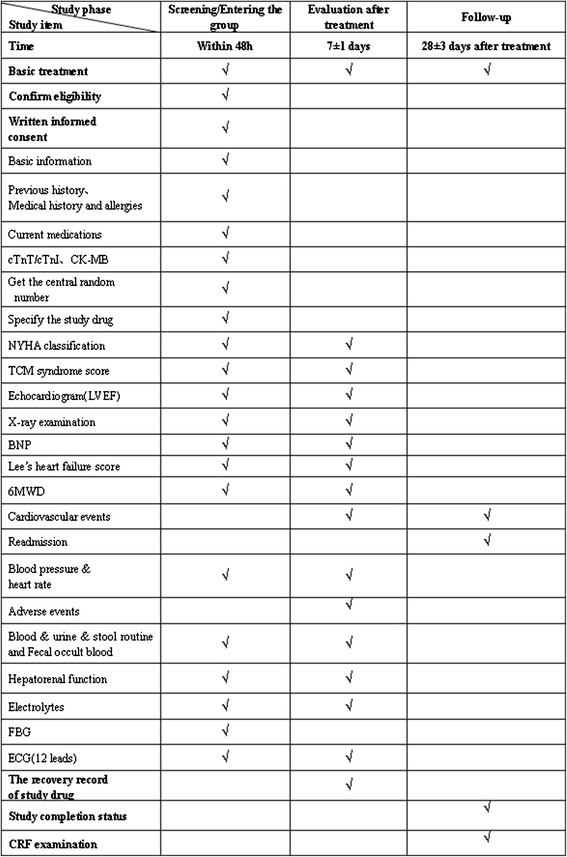
**The trial flow chart.** The work at each point of time is listed. cTnT, cardiac troponin T; cTnI, cardiac troponinI; CK-MB, creatine kinase-MB isoenzyme; NYHA, New York Heart Association; TCM, Traditional Chinese Medicine ; LVEF, left ventricular ejection fraction; BNP, brain natriuretic peptide; 6MWD, 6-minute walking distance; FBG, fasting blood glucose; ECG, electrocardiogram; CRF, case report form.

We will collect basic information (gender, age, previous history, medication and so forth), disease severity assessment (heart functional class, TCM syndrome score, Lee’s heart failure score, 6-minute walking distance), and physical and chemical examinations (echocardiogram, BNP and so forth); specific items and study visits are shown in Figure 2.

Data information will be separately filled in the study medical record and the case report form by researchers. The study medical record is designed to be convenient for the researchers. Based on the need for data traceability, the study medical record preserves original information, such as the patient’s name, contact information, the results of physical and chemical examinations and so forth. The design of the case report form is convenient for data entry. The patients are distinguished by code, and their personal and private information will not be shown.

### Statistical analysis

Statistical analysis will be performed by the Tianjin Institute of Clinical Evaluation, and the data will be analyzed using Statistical Analysis System (SAS) software version 9.3 (SAS Institute Inc., Cary, NC, USA). Data sets for efficacy assessment will follow the intention-to-treat principle. Continuous variables will be described using mean and standard deviation and tested by *t* tests. Categorical variables will be presented using percentages and tested by chi-squared test. More details will be described in a formal statistical analysis plan.

### Ethical issues

This trial protocol was approved by the Ethics Committee of the First Teaching Hospital of Tianjin University of TCM (TYLL2012 [K] 003) and other Institutional Review Boards of participating hospitals (the names of all ethical bodies can be found in Additional file 1). The study protocol and informed consent are consistent with scientific and ethical requirements. Written informed consent must be obtained from all participants or their legally authorized representatives before enrollment.

## Discussion

The efficacy of integrative treatment for AECHF with TCM and western medicine has been gradually accredited with improving symptoms, signs, quality of life, exercise tolerance, long-term prognosis (reducing mortality and readmission rate of CHF), and so forth, and has been demonstrated in clinical studies [18,19].

As a post-marketed herbal species used for nearly 30 years, the composition of SFI is clear and its quality is reliable. Some clinical reports showed that the conventional western medicine combined with SFI could further improve the symptoms of CHF patients, TCM syndrome, the quality of life and show a clinical curative effect, increase LVEF and decrease left ventricular end-diastolic diameter [20], reduce plasma BNP and cytokine Fas, tumor necrosis factor-α, and interleukin-6 levels, as well as mobilize the function of bone marrow stem cell [21,22]. Some research revealed that SFI could attenuate post-resuscitation myocardial dysfunction by increasing the enzyme activity on Na^+^-K^+^-ATP and Ca^2+^-ATP of the left ventricle [23], increasing superoxide dismutase and decreasing malondialdehyde, promoting energy metabolism and anti-oxidative damage.

The study design of this trial has three main points. (1) Selection of patients with AECHF due to CHD; this is a specific disease group and a study on the primary cause and the main type of hospitalization. (2) This is a superiority trial comparing a study drug with placebo. In view of the external characteristics of TCM injection, the study uses the method of blind imitation, with an intravenous drip of 5% GLI with a light-proof infusion cover. (3) The effective rate of heart functional class and TCM syndrome are primary outcomes; it will adopt the clinical effect evaluation methods of “the combination of disease and syndrome, systematic staging, multi-dimension index” [24], and will select indices that reflect both the advantages and characteristics of TCM and the conventional efficacy evaluation of western medicine, and observation of the incidence of end-point events. In addition, the drug dosage of the test group was decided upon through literature research [25] and taken to be 50 ml SFI dissolved in 100 ml 5% GLI via intravenous drip within 150 minutes, in order to relieve cardiac load as much as possible.

### Trial status

The trial was initiated in March 2013. Now, the study is in the stage of database testing and is ready to data input and statistics. The completion date of trial is estimated at December 2015.

## Additional file

Additional file 1:
**Names of all the ethical bodies.**

## Abbreviations

CHF: chronic heart failure
AECHF: acute exacerbation of chronic heart failure
AHF: acute heart failure
CHD: coronary heart disease
TCM: Traditional Chinese Medicine
SFI: Shenfu injection
BNP: brain natriuretic peptide
GLI: glucose injection
LVEF: left ventricular ejection fraction
