# Supplementary material for: Clinical assessment of Shenfu injection loading in the treatment of patients with exacerbation of chronic heart failure due to coronary heart disease: study protocol for a randomized controlled trial
Source: Trials. 2015 May 21;16:222. doi: 10.1186/s13063-015-0729-7 (PMC4446800; doi:10.1186/s13063-015-0729-7)
Supplement: Additional file 1: — Names of all the ethical bodies. [file 13063_2015_729_MOESM1_ESM.doc]

**The names of all ethical bodies that approved study**

**in the various centers involved**

1. Independed Ethics Committee(IEC) of The First Affiliated Hospital of Tianjin University of Traditional Chinese Medicine

**Note:** The First Affiliated Hospital of Tianjin University of Traditional Chinese Medicine is head unit.

2. [Ethics committee](http://dict.cnki.net/dict_result.aspx?searchword=伦理委员会&tjType=sentence&style=&t=ethics+committee) of The Second Affiliated Hospital of Tianjin University of Traditional Chinese Medicine

3. [Ethics committee](http://dict.cnki.net/dict_result.aspx?searchword=伦理委员会&tjType=sentence&style=&t=ethics+committee) of Xiyuan Hospital China Academy of Science of TCM

4. [Ethics committee](http://dict.cnki.net/dict_result.aspx?searchword=伦理委员会&tjType=sentence&style=&t=ethics+committee) of PLA General Hospital

5. [Ethics committee](http://dict.cnki.net/dict_result.aspx?searchword=伦理委员会&tjType=sentence&style=&t=ethics+committee) of Shuguang Hospital Affiliated to Shanghai University of TCM

6. [Ethics committee](http://dict.cnki.net/dict_result.aspx?searchword=伦理委员会&tjType=sentence&style=&t=ethics+committee) of The First Hospital Affiliated to Guangzhou University of TCM

7. [Ethics committee](http://dict.cnki.net/dict_result.aspx?searchword=伦理委员会&tjType=sentence&style=&t=ethics+committee) of West China Hospital Sichuan University

8. Ethics Committee of Sichuan Regional on Chinese Medicine

9. [Ethics committee](http://dict.cnki.net/dict_result.aspx?searchword=伦理委员会&tjType=sentence&style=&t=ethics+committee) of The First Hospital Affiliated to Guangxi University of TCM

10. [Ethics committee](http://dict.cnki.net/dict_result.aspx?searchword=伦理委员会&tjType=sentence&style=&t=ethics+committee) of Ruikang Hospital Affiliated to Guangxi University of TCM

11. [Ethics committee](http://dict.cnki.net/dict_result.aspx?searchword=伦理委员会&tjType=sentence&style=&t=ethics+committee) of Affiliated Hospital of Jiangxi Medical Colleage of TCM
